# Mental and somatic comorbidity of depression: a comprehensive cross-sectional analysis of 202 diagnosis groups using German nationwide ambulatory claims data

**DOI:** 10.1186/s12888-020-02546-8

**Published:** 2020-03-30

**Authors:** Annika Steffen, Julia Nübel, Frank Jacobi, Jörg Bätzing, Jakob Holstiege

**Affiliations:** 1 Central Research Institute of Ambulatory Health Care in Germany (Zi), Berlin, Germany; 2 Department of Epidemiology and Health Monitoring, Unit 26 Mental Health, Robert Koch Institute, Berlin, Germany; 3 Psychologische Hochschule Berlin, Berlin, Germany

**Keywords:** Ambulatory claims data, Comorbidity, Depression, Mental disorders, Somatic diseases

## Abstract

**Background:**

Depression is frequently accompanied by other mental disorders and various somatic diseases; however, previous comorbidity studies often relied on self-reported data and have not simultaneously assessed the entire spectrum of mental and somatic diagnoses. The aim is to provide a complete picture of mental and somatic comorbidity of depression in routine outpatient care in a high income country with a relatively well equipped health care system.

**Methods:**

Using ambulatory claims data covering 87% of the German population (age 15+), we designed a cross-sectional study by identifying persons diagnosed with mild, moderate and severe depression in 2017 (*N* = 6.3 million) and a control group matched 4:1 on sex, 5-year age group and region of residence (*N* = 25.2 million). Stratified by severity, we calculated the prevalence of 202 diagnosis groups included in the ICD-10 in persons with depression as compared to matched controls using prevalence ratios (PR).

**Results:**

Nearly all mental disorders were at least twice as prevalent in persons with depression relative to controls, showing a dose-response relationship with depression severity. Irrespective of severity, the three most prevalent somatic comorbid diagnosis groups were ‘other dorsopathies’ (M50-M54), ‘hypertensive diseases’ (I10-I15) and ‘metabolic disorders’ (E70-E90), exhibiting PRs in moderate depression of 1.56, 1.23 and 1.33, respectively. Strong associations were revealed with diseases of the central nervous system (i.e. multiple sclerosis) and several neurological diseases, among them sleep disorders, migraine and epilepsy, most of them exhibiting at least 2- to 3-fold higher prevalences in depression relative to controls. Utilization of health care was higher among depression cases compared to controls.

**Conclusions:**

The present study based on data from nearly the complete adolescent and adult population in Germany comprehensively illustrates the comorbidity status of persons diagnosed with depression as coded in routine health care. Our study should contribute to increasing the awareness of the strong interconnection of depression with all other mental and the vast majority of somatic diseases. Our findings underscore clinical and health-economic relevance and the necessity of systematically addressing the high comorbidity of depression and somatic as well as other mental diseases through prevention, early identification and adequate management of depressive symptoms.

## Background

Major depressive disorder (MDD) is one of the most common mental disorders worldwide with a lifetime risk of 15–18% [1, 2]. Roughly 7% of the general population experiences a depressive episode within a 12-month period [1, 3, 4]. Depression severely limits psychosocial functioning and quality of life and ranks as a leading cause of disease burden worldwide [5, 6]. With an increased mortality risk of 50%, depression is a risk factor comparable in strength to smoking [7–9].

A large body of evidence from epidemiological surveys has documented a strong interconnection of depression with other mental disorders, most notably with anxiety disorders and substance use disorders [10, 11]. Approximately, 50–60% of individuals with a lifetime history of depression also report a lifetime history of at least one anxiety disorder [11, 12]. Results from a large US survey showed that 14% of respondents with MDD in the prior 12 months also had an alcohol use disorder and 4.6% had a drug use disorder [13]. Among those with lifetime MDD, 40% had an alcohol use disorder and 17% had a drug use disorder [13]. The presence of psychiatric comorbidity in depression is associated with greater severity (e. g. suicidality [14]), as well as slower recovery, higher risk of chronicity, recurrence, treatment resistance [15, 16], and increased utilization of medical services compared to “pure” depression [11, 12, 17].

Depression has been further identified as independent risk factor and negative prognostic factor for many chronic somatic disorders, including diabetes, cardiovascular disease, hypertension, chronic respiratory disorders, and arthritis [18–34]. Comorbid depression in physical disease is related to poor quality of life, worse course of the physical disorder, higher functional impairment and disability, increased service utilization and higher medical costs, and increased mortality compared to the presence of either depression or the physical disease alone [18, 20, 24, 25, 27–38].

Data from the US demonstrate that comorbidities account for the largest portion of the growing economic burden of depression and highlight the importance of considering comorbidities in the treatment of depression [30]. This, however, requires empirical evidence on the relative importance of individual comorbidities in depression. Previous studies investigating the co-occurrence of depression with other mental or somatic disorders have usually focused on single or a few, mainly common, diseases and assessed these comorbidities via self-report [1, 28, 29, 38–41]. In addition, a comprehensive quantification of the excess risk for a broad range of comorbid diseases in individuals with depression relative to persons without depression is currently lacking. On a large-scale basis, the present comparison allows identifying comorbidities based on actually coded medical diagnoses that a) are of high prevalence among individuals with depression and/or b) exhibiting a high excess risk in comparison to persons without depression and, thereby, may ultimately contribute to an improved medical care of persons with depression. Finally, the associations with specific comorbidities may differ according to severity of depression, though a differentiated view on the population level is currently lacking.

Given their size and nearly full coverage, administrative data offer the unprecedented opportunity to simultaneously investigate the association between unipolar depression (MDD or Dysthymic Disorder [DD]) and a large number of mental and somatic disorders. Using claims data from 87% of the German population, the present study set out to simultaneously quantify associations of depression with 202 diagnosis groups included in the ICD-10 as coded by physicians and other mental health professionals. This allows identifying a) the most prevalent mental and somatic comorbidities among individuals with depression as well as b) identifying those comorbidities exhibiting the highest excess risk in depression relative to controls without depression. Such a comprehensive evaluation of the comorbidity in depression at the population level within one study is fundamental to understanding the size and nature of the health challenges posed by depression.

## Methods

### Data source

The present analysis was based on nationwide statutory health insurance (SHI) physicians’ claims data from the years 2009 to 2017, with 2017 being the reporting year. The data cover all SHI insurants in Germany amounting to roughly 70 million individuals and reflecting approximately 87% of the total German population. No data were available for residents with private health insurance (~ 13% of the German population). Among others, the present data source includes information on the insurant’s age, sex, residential area and on all diagnoses documented in the ambulatory care setting according to the ICD-10.

### Study design and study population

We designed a case-control study focusing on patients with a diagnosis of unipolar depression (F32x, F33x or F34.1) in the year 2017 aged ≥15 years. Cases of depression were categorized into mild, moderate and severe based on the documented diagnostic code according to the ICD-10 (Table 1).

**Table 1:** Description of included diagnostic F-codes and their classification according to severity

Severity	ICD-10	Description
Mild	F32.0	Mild depressive episode
	F33.0	Recurrent depressive disorder, current episode mild
	F34.1	Dysthymia
Moderate	F32.1	Moderate depressive episode
	F33.1	Recurrent depressive disorder, current episode moderate
Severe	F32.2	Severe depressive episode without psychotic symptoms
	F32.3	Severe depressive episode with psychotic symptoms
	F33.2	Recurrent depressive disorder, current episode severe without psychotic symptoms
	F33.3	Recurrent depressive disorder, current episode severe with psychotic symptoms
Unspecified/ Other	F32.8	Other depressive episodes
	F32.9	Depressive episode, unspecified
	F33.8	Other recurrent depressive disorders
	F33.9	Recurrent depressive disorder, unspecified
	F33.4	Recurrent depressive disorder, currently in remission

**Fig. 1:**
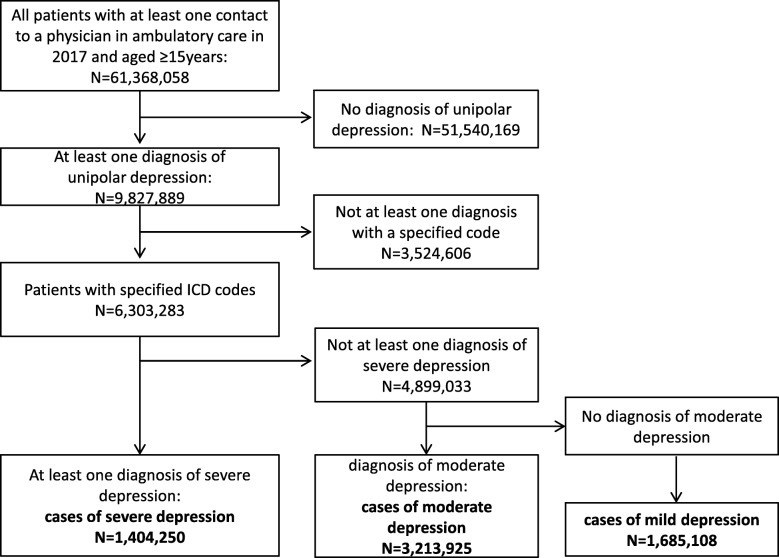
Selection of study participants. A hierarchical classification, considering only the most severe diagnosis, was used to define cases of mild, moderate and severe depression based on diagnostic codes documented in ambulatory care. From the group of all persons with a least one diagnosis of depression in 2017 (*n* = 9,827,889), cases of severe depression were defined as patients with at least one diagnosis of F32.2, F32.3, F33.2 or F33.3 (*n* = 1,404,250). From the remaining patients, those who had at least one diagnosis of F32.1 or F33.1 were classified as cases with moderate depression (*n* = 3,213,925). Finally, patients with a diagnosis of F32.0, F33.0 or F34.1 were classified to have mild depression (1,685,108). Patients who did not have at least one specific diagnostic code of depression to differentiate the severity of the disease were not included in the present analysis (*n* = 3,524,606). Thus, cases exclusively coded with F32.8, F32.9, F33.8 and F33.9 were not considered as cases in the present study

As illustrated in Fig. 1, we used a hierarchical classification and considered only the most severe diagnosis. In brief, from all persons with a least one diagnosis of depression in 2017 (*n* = 9,827,889), cases of severe depression were defined as patients with at least one diagnosis of F32.2, F32.3, F33.2 or F33.3 (*n* = 1,404,250). From the remaining patients, those who had at least one diagnosis of F32.1 or F33.1 were classified as cases with moderate depression (*n* = 3,213,925). Finally, patients with a diagnosis of F32.0, F33.0 or F34.1 were classified to have mild depression (1,685,108). Patients who did not have at least one specific diagnostic code of depression to differentiate the severity of the disease were not included in the present analysis (*n* = 3,524,606). Thus, cases exclusively coded with F32.8, F32.9, F33.8 and F33.9 were not considered as cases in the present study. Depressive syndromes within other ICD-10 categories beyond F3.x (e.g., in organic mental disorders, adjustment disorder with depressive features, mixed anxiety and depression, depressive syndromes in Parkinson’s disease, dementias, or stroke) were not considered as depression cases but as comorbidities.

A control group of persons without depression matched by age (5-year categories), sex and residential area (17 regions representing the different Associations of Statutory Health Insurance Physicians in Germany) was drawn separately for each subgroup of cases (mild, moderate and severe) with a case-control ratio of 1:4. To be included as a control, insurants had to be a) free of any diagnosis of moderate and severe depression during the whole observation period (2009–2017) and b) free of any diagnosis of mild and unspecified depression during the preceding 4 years (2014–2017). This relaxed criterion with respect to previous diagnoses of mild and unspecified depression was used to account for our observation that within specific age and sex strata it appears rather uncommon to not have received such a diagnosis at least once during a period of 9 years.

### Selection of comorbid mental and somatic diseases

The present analysis was based on 202 diagnosis groups stemming from 18 chapters of the ICD-10, excluding diagnostic groups from chapters XVI (‘Certain conditions originating in the perinatal period’; P00-P96), XVIII (‘Symptoms, signs and abnormal clinical laboratory findings, not elsewhere classified’; R00-R99), XXI (‘Factors influencing health status and contact with health services’; Z00-Z99) and XXII (‘Codes for special purposes’; U00-U85).

### Statistical analysis

Characteristics of the study population with regard to sex, age and service utilization in 2017 are reported by depression severity. To describe outpatient service utilization, we used the frequency of contact to ambulatory health care in 2017 as measured by a) the number of different doctors consulted, b) the number of diagnosis groups reflecting the number of different diagnoses a person has received, and c) the number of treatment cases that were generated. In German SHI data, a treatment case is defined on the patient-level as the sum of all medical services delivered by one physician during one quarter. Lastly, we computed the total cost of ambulatory care as the sum of costs related to all medical services that were utilized in ambulatory care in 2017.

Associations of depression with other mental and somatic diseases (202 diagnosis groups) were quantified by computing prevalence ratios (PR), defined as the ratio of the prevalence among depression cases to the prevalence among controls. Results of all investigated comorbidities were presented separately for mental and somatic diseases. Given the high number of somatic diseases included and in order to depict their clinical and public health relevance, PRs for comorbid somatic conditions are displayed in scatterplots across the range of the comorbidity prevalence among controls. Further, a ranking of the 20 most prevalent comorbidities (mental and somatic combined) is presented to illustrate the global picture of relevant comorbidities based on their occurrence. For the sake of completeness, we additionally provide a supplemental table presenting information on all included disease groups, their description according to ICD, the respective ICD chapter they belong to and the prevalence among cases as well as the corresponding prevalence ratio stratified by depression severity (Appendix table A1).

Comorbidity associations were examined by severity of depression in the total study population and for men and women separately. All analyses were conducted using SAS®, version 9.4.

## Results

### Sociodemographic characteristics and service utilization of the study population

We identified 6,303,283 patients with a specific diagnosis of depression in 2017. According to severity, 27% (*N* = 1,685,108) had mild depression, 51% (*N* = 3,213,925) moderate and 22% (*N* = 1,404,250) severe depression. Characteristics of the study population are presented in Table 2. Mean age of the study population was 55 years and two thirds were women. Depression cases showed a higher utilization of medical services than controls, as assessed by several parameters. In brief, patients with depression consulted a higher number of different doctors than controls, generated more treatment cases and were more likely to have received diagnoses from a larger number of diagnosis groups relative to control persons (e.g. more than threefold multimorbid cases with > 20 diagnostic codes). Ultimately, the higher health care utilization of depression cases was reflected in more than two-fold higher treatment costs compared to controls in the ambulatory setting.

**Table 2:** Characteristics of the study population according to severity of depression diagnosis, 2017

	mild depression		moderate depression		severe depression	
	cases	controls	cases	controls	cases	controls
Number of insurants	1.685.113	6.740.452	3.213.925	12.855.700	1.404.250	5.617.000
Age (mean, SD)	56.1 (18.8)	56.1 (18.8)	55.4 (17.8)	54.3 (17.9)	55.5 (17.2)	55.4 (17.3)
Women (%)	67.4	67.4	67.8	67.8	65.3	65.3
Number of different doctors seen in 2017 (median, Q1, Q3)	8 (5, 12)	5 (3, 8)	8 (5, 12)	5 (3, 8)	8 (5, 12)	5 (2, 8)
Number of diagnosis groups (%)						
≤ 10 diagnosis groups	43	69	44	71	41	70
11- ≤ 20 diagnosis groups	41	26	40	24	41	25
21+ diagnosis groups	16	5	16	5	18	5
Number of treatment cases in 2017 (median, Q1, Q3)	12 (8, 17)	8 (4, 12)	12 (8, 18)	7 (4, 12)	13 (9, 19)	7 (4, 12)
Total cost of ambulatory care (€) in 2017 (median, Q1, Q3)^a^	724 (393, 1248)	357 (155, 684)	832 (448, 1484)	340 (148, 652)	892 (495, 1552)	345 (150, 661)

*SD* Standard deviation, *Q1* Quartile 1, *Q3* Quartile 3

^a^Costs of ambulatory drug prescriptions are not included

### Mental comorbidity

**Fig. 2:**
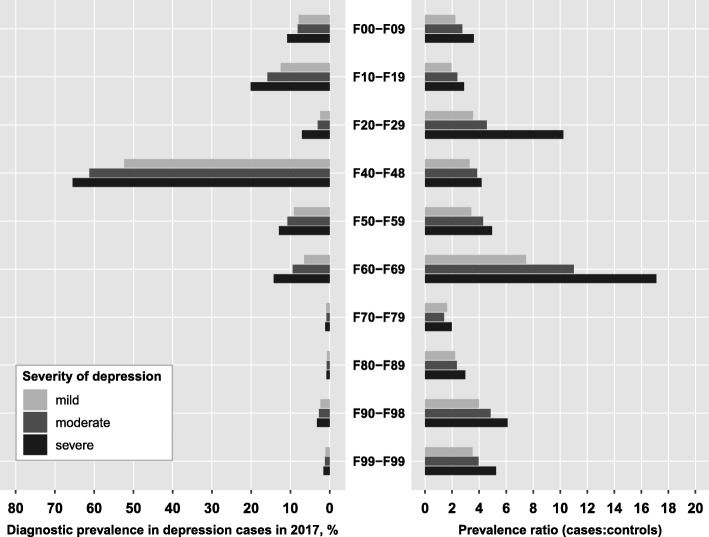
Prevalence of mental disorders among cases with unipolar depression and prevalence ratios relative to controls. Diagnosis groups according to ICD-10: F00-F09, Organic mental disorders; F10-F19, Substance use disorders; F20-F29, Schizophrenia; F40-F48, Neurotic, stress-related and somatoform disorders; F50-F59, Behavioral syndromes associated with physiological disturbances and physical factors; F60-F69, Disorders of adult personality and behavior; F70-F79, Mental retardation; F80-F89, Disorders of psychological development; F90-F98, Behavioral and emotional disorders with onset in childhood and adolescence; F99-F99, Unspecified mental disorders. The prevalence ratio is defined as the ratio of the prevalence of the respective diagnosis group among depression cases to the prevalence among age-, sex- and region-matched controls

Overall, 64% of mild depression cases (72% of moderate, 78% of severe) had a comorbid mental disorder. Figure 2 presents the prevalence of diagnosed mental disorders in depression cases according to severity of depression and the prevalence ratio of the respective comorbidity relative to controls. In general, the prevalence of comorbid disorders increased with depression severity, amounting to a 30 to 40% higher prevalence for most disorders in severe compared to mild depression cases. Larger differences were only observed for schizophrenia (3-times and 2-times higher in severe and moderate compared to mild depression cases, respectively). Neurotic, stress-related and somatoform disorders (F4) were by far the most prevalent comorbidity in depression, irrespective of depression severity; 65% of severe depression cases (52% of mild and 61% of moderate cases) had additionally received an F4-diagnosis. The second most frequent psychiatric comorbidity was the group of substance use disorders (F1) which amounted to a diagnostic prevalence of 12, 16 and 20% in mild, moderate and severe cases. Personality disorders (F6) and behavioral syndromes (F5) ranked third and fourth with prevalence values ranging between 6 and 14% (Fig. 2).

Overall, depression cases were at least twice as likely to have a mental comorbidity compared with age- and sex-matched controls, with mental retardation (F7) being the only exception (PR between 1.4 and 2.0). While most mental comorbidities exhibited prevalence ratios between 2 and 6, personality disorders showed strikingly higher PRs (PR = 11 in moderate and PR = 17 in severe depression). In addition, severe depression cases had a 10-fold higher risk of being diagnosed with schizophrenia compared to controls, while the risk of mild and moderate depression cases was substantially lower (PRs of 3.5 and 4.6, respectively, Fig. 2).

### Somatic comorbidity

**Fig. 3:**
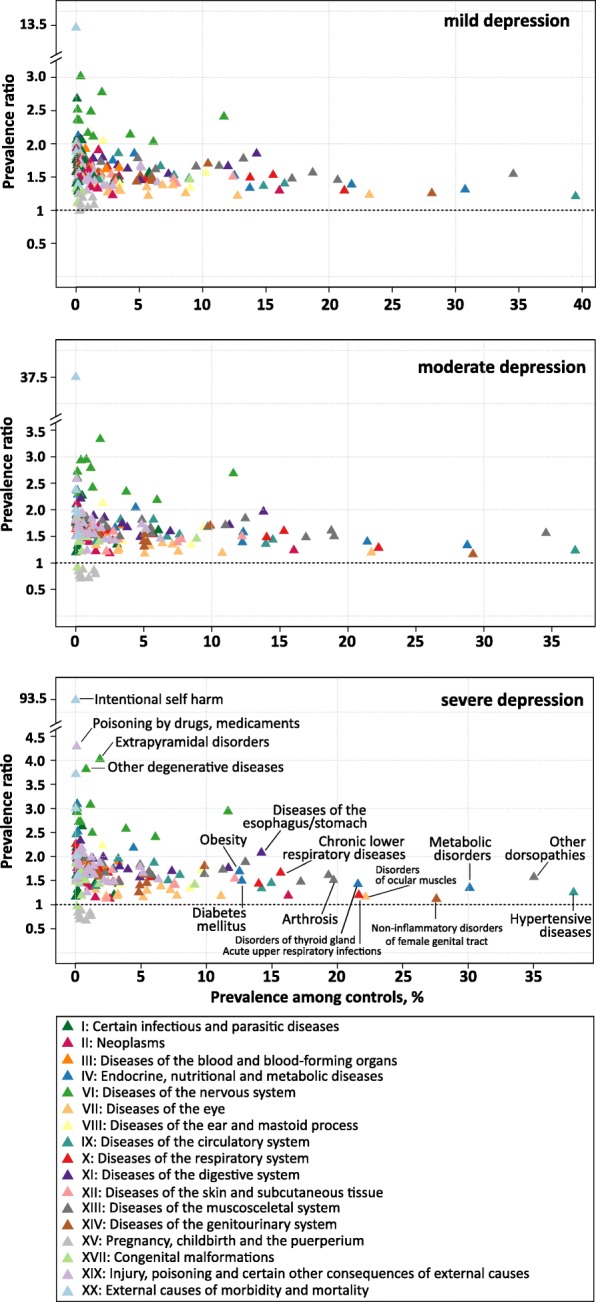
Prevalence ratios of somatic comorbidities according to prevalence among controls by depression severity. Prevalence ratios were estimated for 191 somatic diagnosis groups from the ICD-10 based on administrative data from outpatient care including 6.3 million patients with a specific diagnosis of depression in 2017 and 25.2 million age-, sex- and region-matched controls. The prevalence ratio is defined as the ratio of the prevalence of the respective diagnosis group among depression cases to the prevalence among controls. Diagnosis groups are colored according to the respective chapter of the ICD

The associations between 192 somatic disease groups and depression severity are illustrated in Fig. 3. Almost all somatic diseases were more prevalent among depression cases compared to controls. Exceptions were the group of diagnoses concerning pregnancy, childbirth and puerperium (Chapter XV) which showed an inverse association with moderate and severe depression and no association with mild depression. As already observed for mental disorders, the strength of association gradually increased with severity of depression diagnosis.

Irrespective of severity, the three most prevalent somatic comorbid diagnosis groups were ‘other dorsopathies’ (M50-M54), ‘hypertensive diseases’ (I10-I15) and ‘metabolic disorders’ (E70-E90) (Fig. 3 and Table 3). A total of 54% of moderate depression cases had also received a diagnosis of ‘other dorsopathies’, relating to a 56% higher risk compared to controls (PR = 1.56). In terms of hypertensive diseases and metabolic diseases, moderate depression was related to a 23 and 33% higher risk, respectively (45 and 38% affected moderate depression cases).

Overall, three major groups of somatic comorbidities can be identified in Fig. 3:

First, there is the group of diseases that are relatively common in the general population (prevalence > 10% among controls). This group comprises diseases of the musculoskeletal system (Chapter XIII), endocrine, nutritional and metabolic disorders (Chapter IV) and diseases of the circulatory (Chapter IX) and respiratory system (Chapter X). These prevalent diseases were mainly associated with low to moderate increased risks in depression (PRs between 1.2 and 2.0). In the group of circulatory diseases, ‘Essential (primary) hypertension’ (I10.9) was the single most frequent comorbid diagnosis in moderate depression (38% vs. 31% in controls, PR = 1.2%). Among musculoskeletal diseases, ‘low back pain’ (M54.5) and ‘Cervicalgia’ (M54.2) both affecting 15% of moderate depression cases constituted the most frequent comorbid diagnoses (PR = 1.7 and PR = 2.0, respectively). With regard to endocrine, nutritional and metabolic disorders (Chapter IV), pure hyperchlesterolaemia (E78.0) was the single most frequent diagnosis, affecting 17% of moderate depression cases (PR = 1.3), followed by type 2 diabetes mellitus (E11.9) with a prevalence of 13% (PR = 1.4) and hypothyroidism (E03.9) with a prevalence of 12% (PR = 1.6). Within the group of respiratory diseases, apart from acute upper respiratory infections (J00-J06; *P* = 28%; PR = 1.3), diagnoses of asthma and chronic obstructive pulmonary disease belonged to the group of common diseases (J40-J47), generally displaying a 1.8- to 2.2-fold higher prevalence in depression cases compared to controls.

Second, we observed a cluster of somatic comorbidities that were rather infrequent in the general population (prevalence < 2%, except ‘G40-G47’) and strongly related to the diagnosis of depression, exhibiting prevalence ratios > 2.5. Among mild, moderate and severe cases a total of 4, 9 and 16 disease groups displayed a PR beyond this threshold, predominantly comprising diseases of the nervous system (Chapter VI, G.x). In mild depression, the highest PR was observed for demyelinating diseases of the central nervous system (G35-G37; PR = 3.02), mainly reflecting the diagnosis of multiple sclerosis (G35.9 and G35.1). In moderate and severe depression, extrapyramidal and movement disorders (G20-G26; PR of 3.33 and 4.02, respectively) were related to the highest excess risks. The main contributor to this disease group were ‘restless legs syndrome’ (G25.81; prevalence of 2.8% in moderate and of 3.3% in severe depression cases) followed by ‘primary Parkinson disease’ (G20.9) with a prevalence of 1.2% (PR = 3.6) in moderate and 1.5% (PR = 4.3) in severe depression. Additionally, in moderate depression, other degenerative diseases of the nervous system (G30-G32) and demyelinating diseases of the central nervous system (G35-G37) ranked second and third in terms of increased risk (PR of 2.95 and 2.93, respectively). As indicated above, the group of ‘Episodic and paroxysmal disorders’ (G40-G47) stood out in that it was much more frequent among controls (12%) than the other diseases of the nervous system and strongly associated with depression (PR = 2.7), indicating that 31% of moderate depression cases received a diagnosis from this group. The main contributors to this group in terms of prevalence in moderate depression were unspecified sleep disorders (G47.9; *P* = 7.4%; PR = 4.3), migraine (G43.9; *P* = 7.1%; PR = 2.1) and disorders of initiating and maintaining sleep (G47.0; *P* = 5.8%; PR = 5.5). Diagnoses of epilepsy exhibited prevalence ratios between 2.3 and 4.1, with unspecified epilepsy ranking 9th in terms of prevalence in the total group of episodic and paroxysmal disorders (G40.9; *P* = 2.0%; PR = 2.3).

A third cluster of disease groups can be defined as having low to moderate prevalence in the general population (< 10%) and low to moderate increased risk in depression (PRs > 1.0 and < 2.0). The majority of disease groups were comprised in this cluster, amounting to 145 (72%), 134 (66%), and 124 (61%) among mild, moderate and severe depression cases, respectively.

### Ranking of mental and somatic comorbidity

Table 3 provides an integrated view of the 20 most frequent mental and somatic comorbidities in cases with depression - ranked by their prevalence - and their excess risk in comparison to controls. Irrespective of depression severity, neurotic, stress-related and somatoform disorders (F4) emerged as a highly relevant comorbidity (rank 2 in mild depression, rank 1 in moderate and severe depression). With the exception of substance use disorders that ranked 19th in the group of severe depression cases, all remaining top 20 comorbidities were of somatic nature. While the set of identified comorbidities was largely the same for mild, moderate and severe depression, the ranking slightly differed for some of the comorbidities. For instance, while episodic and paroxysmal disorders (G40-G47) ranked 10th in mild depression (prevalence of 28%), they ranked 6th in moderate (prevalence of 34%) and 5th in severe depression (prevalence of 34%). As a result, the prevalence ratio increased with severity from 2.4 in mild depression to 2.9 in severe depression.

Within control persons, the group of neurotic, stress-related and somatoform disorders (F4) was the only mental comorbidity among the 20 most prevalent comorbidities, ranking 13th (controls of moderate depression cases) or 14th place (controls of mild and severe depression) with a prevalence of nearly 16% (see Table 2). The three most prevalent disorders among controls were hypertension (I10-I15) followed by other dorsopathies (M50-M54) and metabolic disorders (E70-E90), affecting roughly 38, 35 and 30% among control patients, respectively.

**Table 3:** Ranking of the 20 most prevalent mental and somatic comorbid conditions among cases with depression according to severity of diagnosis

mild depressive disorder					moderate depressive disorder					severe depressive disorder				
Rank among cases	Rank among controls	ICD group	prevalence among cases (%)	PR	Rank among cases	Rank among controls	ICD group	prevalence among cases (%)	PR	Rank among cases	Rank among controls	ICD group	prevalence among cases (%)	PR
1	2	M50-M54	53.3	1.54	1	12	F40-F48	61.2	3.84	1	13	F40-F48	65.5	4.18
2	14	F40-F48	52.4	3.29	2	2	M50-M54	53.9	1.56	2	2	M50-M54	55.1	1.57
3	1	I10-I15	47.7	1.21	3	1	I10-I15	45.3	1.23	3	1	I10-I15	47.7	1.25
4	3	E70-E90	40.4	1.31	4	4	E70-E90	38.4	1.33	4	3	E70-E90	40.6	1.35
5	4	N80-N98	35.3	1.26	5	3	N80-N98	34.0	1.16	5	23	G40-G47	34.2	2.94
6	6	E00-E07	30.2	1.39	6	22	G40-G47	31.1	2.68	6	9	M70-M79	31.2	1.61
7	9	M15-M19	30.1	1.45	7	9	M70-M79	30.2	1.61	7	4	N80-N98	30.9	1.12
8	10	M70-M79	29.3	1.57	8	7	E00-E07	29.9	1.40	8	7	E00-E07	30.8	1.43
9	5	H49-H52	28.6	1.23	9	5	J00-J06	28.6	1.28	9	8	M15-M19	29.8	1.51
10	24	G40-G47	28.2	2.41	10	8	M15-M19	28.5	1.50	10	16	K20-K31	29.5	2.08
11	7	J00-J06	27.5	1.30	11	17	K20-K31	27.1	1.97	11	12	J40-J47	26.1	1.67
12	17	K20-K31	26.4	1.85	12	6	H49-H52	26.0	1.20	12	6	J00-J06	26.0	1.20
13	11	M20-M25	25.2	1.48	13	10	M20-M25	25.1	1.48	13	5	H49-H52	25.8	1.16
14	15	J40-J47	23.8	1.53	14	13	J40-J47	24.5	1.60	14	10	M20-M25	25.4	1.48
15	20	M45-M49	23.5	1.78	15	18	M45-M49	22.9	1.84	15	18	M45-M49	24.5	1.89
16	12	I30-I52	23.1	1.40	16	15	J30-J39	20.8	1.48	16	14	I30-I52	21.7	1.45
17	13	D10-D36	20.8	1.30	17	14	I30-I52	20.8	1.44	17	20	E65-E68	21.2	1.69
18	18	J30-J39	20.5	1.49	18	11	D10-D36	19.8	1.24	18	22	K55-K64	20.6	1.76
19	16	I80-I89	20.2	1.36	19	19	E65-E68	19.4	1.58	19	38	F10-F19	20.1	2.89
20	23	K55-K64	20.0	1.66	20	23	K55-K64	19.4	1.71	20	17	J30-J39	20.1	1.43

The prevalence ratio is defined as the ratio of the prevalence of the respective diagnosis group among depression cases to the prevalence among controls.

ICD diagnosis groups: D10-D36, Benign neoplasms; E00-E07, Disorders of thyroid gland; E65-E68, Obesity and other hyperalimentation; E70-E90, Metabolic disorders; F10-F19, Mental and behavioural disorders due to psychoactive substance use; F40-F48, Neurotic, stress-related and somatoform disorders; G40-G47, Episodic and paroxysmal disorders; H49-H52, Disorders of ocular muscles; I10-I15, Hypertension; I30-I52, Other forms of heart disease; I80-I89, Diseases of veins, lymphatic vessels and lymph nodes, not elsewhere classified; J00-J06, Acute upper respiratory infections; J30-J39, Other diseases of upper respiratory tract; J40-J47, Chronic lower respiratory diseases; K20-K31, Diseases of oesophagus, stomach and duodenum; K55-K64, Other diseases of intestines; M15-M19, Arthrosis; M20-M25, Other joint disorders; M45-M49, Spondylopathies; M50-M54, Other dorsopathies; M70-M79, Other soft tissue disorders; N80-N98, Noninflammatory disorders of female genital tract.

### Comparison of men and women

**Fig. 4:**
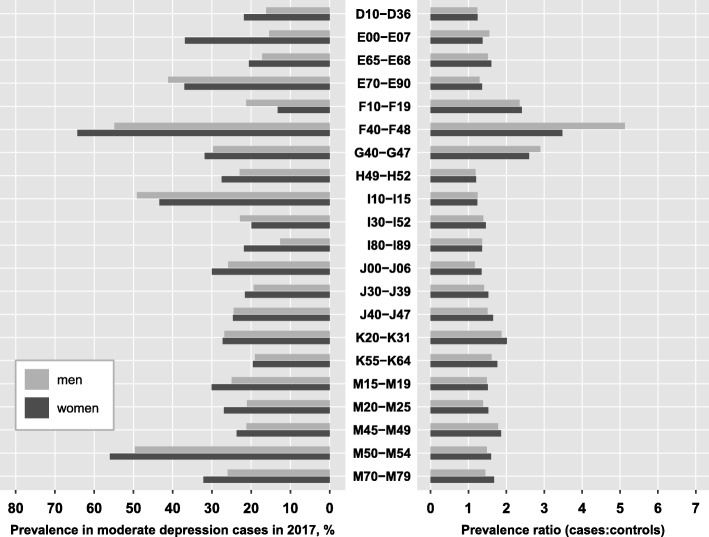
Age-adjusted prevalence and prevalence ratios for the most prevalent comorbidities by sex . Prevalence ratios were estimated for 201 diagnosis groups from the ICD-10 reflecting mental and somatic diseases using administrative data from outpatient care including 6.3 million patients with a specific diagnosis of depression in 2017 and 25.2 million age- and sex-matched controls. Sex-specific prevalence was age-adjusted using the joint age distribution of depression cases as reference, stratified by severity. The prevalence ratio is defined as the ratio of the prevalence of the respective diagnosis group among depression cases to the prevalence among controls

For the most prevalent comorbidities in the total population, we compared the prevalence among depression cases and the corresponding prevalence ratio between men and women. For this comparison, we included all diagnosis groups from Table 3, irrespective of depression severity, excluding N80-N98 as a women-specific diagnosis group. Figure 4 displays the results for moderate depression, while the findings for mild and severe depression can be found in the appendix (Table A2). For the majority of the displayed disease groups, age-adjusted prevalence was higher among female depression cases compared to male cases, except for metabolic disorders (E70-E90), substance use disorders (F10-F19), hypertensive diseases (I10-I15) and other forms of heart disease (I30-I52). Further, the excess risk relative to controls was mainly similar for males and females with depression, with a tendency towards slightly higher risks among women though. A striking exception was observed for the group of neurotic, stress-related and somatoform disorders (F4). Despite the age-adjusted prevalence of this mental comorbidity being substantially lower in men compared to women (55% vs. 65%), the excess risk compared to control persons was 47% higher in men than in women (PR of 5.13 vs. 3.48), implying that male cases of depression are much more likely to additionally have a diagnosis of an F4-disorder compared to controls than female depression cases. In other words, F4-disorders are generally more prevalent among women than men (18% vs. 11% in female and male control persons), but prevalence in men increases more drastically when they are diagnosed with depression.

Ranking of the comorbidities according to their prevalence among depression cases was largely similar between men and women. A few striking differences should be noted though. First, substance use disorders ranked 13th in moderate depression and 10th in severe depression among men, while they ranked 29th and 26th among women, respectively. Despite a comparable prevalence ratio, they were 50 to 60% more likely to occur in men with depression compared to women. Second, diabetes mellitus (E10-E14) which did not reach the top 20 most prevalent comorbidities in the total population (Table 3), came up to rank 16 among men (prevalence: 20.5%), while it ranked only 25th in women (prevalence: 15.4%). Third, other forms of heart disease (I30-I52) ranked substantially higher among men compared to women (i.e. 11th and 22nd in severe depression), despite a comparable prevalence among depression cases (23.5% in men, 20.8% in women).

## Discussion

To our knowledge, the present study is the first to comprehensively illustrate the comorbid status of persons diagnosed with depression with regard to the entire spectrum of diseases diagnosed by medical professionals in outpatient care (i.e. not assessed via self-report such as in the World Mental health surveys [29]). With our unique approach of a) simultaneously investigating about 200 diagnosis groups, while b) differentiating analyses according to depression severity and c) quantifying the excess risk of comorbidity in depression relative to controls based on data from nearly the complete adolescent and adult population in a high-income country with a well-equipped health care system, we were able to generate an overarching picture of the distribution and implications of depression comorbidity for the individual and public health in high-income countries.

### Mental comorbidity

The present study confirms the strong association of depression with anxiety and substance use disorders, as described previously [10, 40, 41]. It further illustrates that nearly all mental disorders are at least twice as likely in persons with depression relative to controls and that excess risk increases with depression severity. Overall, about two thirds of depression cases had at least one other concomitant mental disorder which corresponds well with findings from population surveys documenting that 60–65% of persons with 12-month MDD have at least one other mental comorbid disorder [10, 42–44].

In line with results from international epidemiological surveys in high-income countries [10, 42, 44, 45], we identified neurotic, stress-related and somatoform disorders, which mainly comprise anxiety disorders, as the most frequent mental comorbidity, affecting between 60 and 65% of moderate and severe depression cases. This highly frequent comorbidity has been explained based on a close relation in terms of genetics [46], shared environmental risk factors (i.e. childhood adversities and negative life events [47]) and overlap in symptoms. A recent study further underscored the close relation of both disorders by documenting that MDD, as a narrowly defined episodic disorder, may lead to an underestimation of both the prognosis of the majority of patients and the appropriate type of care [48]. In that large prospective study, 32% of MDD patients appeared to be fully recovered after a follow-up of 6 years when considering only MDD, while this proportion reduced to 17% when taking into account symptoms of anxiety, suggesting that the majority of patients suffer from chronic disorders and that full recovery is the exception. In concordance with European and US population surveys in the general population [49] and also with regard to comorbid anxiety [45], we observed a female preponderance of anxiety disorders in depression, though in men we observed a substantially higher risk for anxiety disorders when relative to their healthy counterparts than in women. This strikingly higher comorbidity risk observed in men needs further investigation; though we hypothesize it might be artefactual. It is well documented that men are less likely 1) to seek help for mental health problems than women, i.e. related to conformity to traditional gender roles, 2) to have symptoms fitting standard measurement tools and 3) to have their mental health problem identified by primary care physicians, all promoting an underestimation of the prevalence, particularly with regard to depression and anxiety disorders [50, 51]. Taking these observations into account, it seems likely that the prevalence of anxiety disorders in the male control group may be underestimated in the present study and/or that depression without anxiety was underdiagnosed in men, both artificially increasing the prevalence ratio. In relation to this, we recently observed that the prevalence of diagnosed depression increased more strongly in (young) men compared to women between 2009 and 2017, proposing a continuous reduction of the gender difference in prevalence of diagnosed depression [52].

Substance use disorders emerged as the second most prevalent mental comorbidity in depression, affecting between 12 and 20% of depression cases, depending on severity of depression. Previous population studies not differentiating between severity status yielded estimates ranging between 9% in the US [10], 13% in New Zealand [44], 17% in the Netherlands [43], and 18% in Australia [45].

With regard to personality disorders, our findings deviate from previous studies that were mainly based on structured interviews using the DSM-system [53, 54]. First, the prevalence of diagnosed comorbid personality disorders observed in our study is strikingly lower than determined in a recent meta-analysis where 30–50% of MDD patients had a comorbid DSM-diagnosed personality disorder [53]. Second, the prevalence of personality disorders in the general population is estimated to be about 9% [54], suggesting a population prevalence as high as observed in our depression cases and much higher than in our control group. Thus, our study supports the general notion of differences in the identification of personality disorders between the DSM-system and ICD-system, with a substantial underdiagnosis in the ICD [53, 55]. The artificial dichotomy that requires clinicians to decide on whether a person does or does not have a personality disorder has been made responsible for the observation that in most cases, the diagnosis is avoided [56]. The 11th version of the ICD has now addressed the need for a dimensional diagnostic approach and allows for classifying different degrees of severity which may ultimately improve identification of personality disturbances in clinical practice. It will be interesting to evaluate this aspect in future studies using administrative health data.

### Somatic comorbidity

By taking into account the whole range of somatic disease groups, we were able to provide an overall picture of somatic comorbidity in depression, including a ranking of the comorbidities according to their prevalence as well as to their excess risk relative to controls. Thereby, our study adds to the existing literature that has already generated compelling evidence on comorbid associations with depression for a large number of somatic diseases, but usually focused on single or a few common diseases at the same time. In general, our findings on the cross-sectional associations of diagnosed depression and comorbid somatic disorders in the ambulatory setting are in large agreement with previous evidence on associations with various somatic diseases, including cardiovascular diseases [29, 57], metabolic diseases [22, 32, 58], neurological diseases [31, 59, 60], cancer [61], immune-mediated inflammatory diseases [62–65], chronic lower respiratory diseases [34, 66] and musculoskeletal diseases [67].

In the following, we discuss some of our findings in the context of previous research by focusing on the very common diseases and high-risk disease groups as described in relation to Fig. 3.

#### Depression and common diseases

We found depression to be associated with several common somatic illnesses, including diseases of the circular system (i.e. hypertension, heart disease and diseases of veins), endocrine, nutrition and metabolic diseases (i.e. diabetes mellitus, obesity) as well as muscoloskeletal diseases (i.e. low back pain, cervicalgia). In general, individuals with depression were 20–60% more likely to additionally have a diagnosis of one of these diseases compared to individuals without depression.

Within this set of relatively common diseases, the group of ‘other dorsopathies’, reflecting low back pain and cervicalgia, was the most frequent diagnosis group in depression cases and, at the same time, was related to the highest risk compared to controls. A significant comorbidity of depression and (chronic) back pain has been observed previously in cross-sectional population surveys in Germany [33] and Canada [68] and has been linked to significant increases in sick days and doctor visits [33] as well as substantially higher direct health care costs [69]. Depression has been shown to increase risk of future low back pain [70], to have a negative impact on pain severity and perception in patients with low back pain [71], and to relate to an impaired quality of life, increased disability and higher risk of chronicity [29, 72].

Hypertensive diseases, as a leading risk factor for morbidity and mortality in the general population, ranked second among depression cases in terms of prevalence and related to a 20% higher risk compared to individuals without depression. In addition, further diseases of the cardiovascular system are found within the top 20 of the most prevalent diseases in depression. Our findings are supported by a large body of evidence documenting associations of depression with incident hypertension [73–75] as well as the incidence and poor prognosis of other cardiovascular diseases, including stroke [18, 76], coronary heart disease [18, 77], and heart failure [57]. Similarly, for various endocrine and metabolic disorders such as hypercholesterolaemia [78], obesity [79] and diabetes mellitus [22, 32], all of which risk factors for cardiovascular diseases, some cancers and overall mortality, associations with depressions have been documented.

#### Depression and high-risk comorbidities

In line with previous literature [31, 59, 63, 80, 81], the present study revealed strong associations of depression with diseases of the central nervous system (i.e. multiple sclerosis) and with several neurological diseases, among them sleep disorders, migraine and epilepsy, most of them exhibiting at least 2- to 3-fold higher prevalence in depression relative to controls. Results of a meta-analysis on depression and epilepsy indicate that 23% of persons with epilepsy also have a depression [80], though studies suggest depression to be underrecognized in individuals with epilepsy [82]. With regard to multiple sclerosis, a Canadian study based on administrative health data has recently estimated the incidence of depression to be 2.4-fold higher in individuals with multiple sclerosis compared to their healthy counterparts [81]. Further, the authors demonstrated a greater than additive interaction of multiple sclerosis with depression on mortality risk, implying that 13% of the increased mortality in multiple sclerosis being due to the joint effects of having multiple sclerosis and depression [63].

Epidemiological studies on the association of migraine and depression have shown a 50% higher risk of depression in persons with migraine and, among persons with depression, a 1.6–3.4-times higher risk for developing migraine [59].

#### Depression and pregnancy

In contrast to all other diagnosis groups, pregnancy-related diagnosis groups were inversely related to moderate and severe, but not mild, depression in our study. A range of cross-sectional studies has previously suggested that depression may be associated with decreased fecundability, though the temporal sequence of events remained unclear [83]. A recent prospective internet-based preconception cohort study of women attempting to conceive recently suggests that moderate to severe depressive symptoms at baseline, independent of psychotropic medication use, were related to decreased fecundability compared to no or low depressive symptoms [84].

### Biological mechanisms linking depression to comorbid diseases

The high comorbidity of depression with a broad range of somatic diseases may reflect various mechanisms. First, depression is associated with unhealthy behavior, i.e. smoking, alcohol consumption, lack of physical activity, poor diet, and impaired sleep, all well-established risk factors for common chronic diseases such as diabetes and cardiovascular diseases [25]. In addition, depression has been associated with non-adherence to treatment regimens, which may explain the worse prognosis among patients with somatic disease and comorbid depression [85]. Second, depression has been shown to have neuroendocrine effects, i.e. the activation of the sympathetic nervous system and dysregulation of the hypothalamic-pituitary-adrenal (HPA) axis which among others promotes endothelial dysfunction, hypertension, abdominal obesity, hypercholesterolemia and hypertriglyceridemia, conferring higher risks of diabetes and cardiovascular diseases [18, 85]. Also, elevated HPA activity may impact menstrual cycle characteristics, subsequently affecting the ability to conceive [86]. Third, accumulating evidence indicates that depression is related to a state of chronic low-grade inflammation, with significantly increased levels of interleukin (IL)-1, IL-6, tumor-necrosis factor (TNF)-alpha and C-reactive protein (CRP). The role of immune-mediated inflammation is increasingly recognized as the universal pathophysiological process that underlies numerous somatic diseases (diabetes, stroke, heart disease, many cancers, autoimmune diseases such as multiple sclerosis and rheumatoid arthritis) as well as mental disorders, including depression [18, 65]. Based on these emerging observations on shared biological mechanisms involved, the association of depression with various somatic diseases (i.e. coronary heart disease, stroke, migraine, chronic obstructive pulmonary disease, rheumatoid arthritis) is likely bidirectional, with abnormalities present in depression increasing the risk of the somatic disease and the presence of the somatic disease or its determinants contributing to the development of depression [18, 25, 59, 65, 66, 87, 88]. It is increasingly understood that the inflammatory processes in individuals with depression and a comorbid somatic disease together with neuroendocrine effects and behavioral factors associated with depression all feed off each other in a self-perpetuating feedback loop, affecting development, severity, prognosis and outcome of both disorders [18, 87]. A recent study using Mendelian randomization to elucidate shared mechanisms underlying the association of depression and coronary heart disease provided evidence that triglycerides, IL-6 and CRP are causally related to depression [89]. Using a similar approach with regard to obesity, studies indicate that obesity is a causal risk factor for depression, but not vice versa, with a recent study specifying body fat mass to be the driver of this causal relationship [58].

### Implications for clinical practice and public health

As key finding, our study underscores the importance of closely surveying symptoms associated with depression in chronic somatic diseases in primary care and, conversely, considering the increased risk of somatic diseases, i.e. cardiometabolic disturbances, related to depression. The results of our study support the development of interdisciplinary and multidisciplinary treatment strategies, integrating mental health services into primary care, which have been shown to improve treatment adherence, outcomes and quality of life [90]. This may eventually require changes in the organization of the health care system, i.e. including the establishment of a regular and standardized screening for depression in primary care, adjustment of training of health workers in the primary care with regard to the frequent comorbidity of depression and somatic disorders and inclusion of mental health workers, improved referral to qualified health care provider, harmonization of care between healthcare providers, as well as education of the general population and individuals with somatic diseases [25, 32]. As important step towards early detection and improved treatment, some of the latest treatment guidelines for common somatic diseases have now included recommendations on screening for depression, i.e. the European guidelines on cardiovascular disease prevention [91] or the American clinical practice recommendations on the management of diabetes [92]. In Germany, the Federal Join Committee (G-BA) has recently issued the implementation of a structured treatment plan for depression (disease management program), defining the adequate treatment of relevant comorbidities as one major treatment target [93, 94].

### Limitations

Limitations of our study refer to the commonly recognized constraints of administrative data for epidemiological research. Results are based on the presence of specific diagnostic codes that were routinely collected for the purpose of physician billing claims. Hence, the validity depends on the accuracy of those codes and studies have shown validity to vary by disease [95].

Given the cross-sectional design, it is not possible to draw conclusions about causes and effects and interactions of the observed associations. Potential pathways include a) the contact to the health care system is more pronounced among persons with depression compared to persons without depression, which increases the likelihood of early detection and diagnosis of further existing diseases in depression cases; b) the presence of (somatic) diseases or its medical treatment may lead to the development of depressive symptoms and similarly increase the likelihood of the diagnosis; c) for some diseases, the typical symptoms overlap with or mimic the specific symptoms of depression (i.e. hypothyroidism); d) further, enhanced comorbidity may be due to shared risk factors and pathological pathways between depression and other conditions.

Finally, the present analysis did not evaluate excess mortality in comorbid cases (as was documented in particular in severe mental disorders, i.e. [96, 97]).

## Conclusion

The present study provides a comprehensive overview of depression comorbidity and should stimulate awareness of the strong interconnection of depression with all other mental and the vast majority of somatic diseases. Our findings underscore the necessity of systematically addressing the high comorbidity of depression and somatic diseases in primary care through prevention, early identification of vulnerable persons and management of depressive symptoms, i.e. within the framework of disease management programs. Given the extensive association with several of the most burdensome somatic diseases and other mental disorders, as well as excess utilization of health care services (which may reflect excess needs), depression has evolved to a central health care problem that requires the integration of interdisciplinary and multidisciplinary treatment strategies.

## Supplementary information

**Additional file 1:Table A1.** Prevalence (%) and prevalence ratio (PR) for all 202 ICD diagnosis groups included in the present study. This supplemental table provides the prevalence and prevalence ratios stratified by depression severity for all 202 included diagnosis groups.

**Additional file 2:Table A2.** Age-adjusted prevalence (%) and prevalence ratios (PR) for the 20 most prevalent comorbidities by sex among individuals with mild depressive disorder. This supplemental table provides age-adjusted prevalence and prevalence ratios for the 20 most prevalent comorbidities by sex among individuals with mild depressive disorder.

## Data Availability

The datasets analyzed during the current study are not publicly available due to ethical and privacy reasons.

## Abbreviations

CRP: C-reactive protein
DD: Dysthymic Disorder
DSM: Diagnostic and Statistical Manual of Mental Disorders
HPA axis: Hypothalamic-pituitary-adrenal axis
ICD: International Classification of Diseases
IL-1: Interleukin-1
MDD: Major Depressive Disorder
PR: Prevalence Ratio
SAS: Statistical Analysis Software
SHI: Statutory Health Insurance
TNF-alpha: Tumor-necrosis factor alpha
