# Supplementary material for: Mental and somatic comorbidity of depression: a comprehensive cross-sectional analysis of 202 diagnosis groups using German nationwide ambulatory claims data
Source: BMC Psychiatry. 2020 Mar 30;20:142. doi: 10.1186/s12888-020-02546-8 (PMC7106695; doi:10.1186/s12888-020-02546-8)
Supplement: Supplementary file 1 — Additional file 1:Table A1. Prevalence (%) and prevalence ratio (PR) for all 202 ICD diagnosis groups included in the present study. This supplemental table provides the prevalence and prevalence ratios stratified by depression severity for all 202 included diagnosis groups. [file 12888_2020_2546_MOESM1_ESM.docx]

Table A1. Prevalence (%) and prevalence ratio (PR) for all 202 ICD diagnosis groups included in the present study

| **Diagnosis group of the ICD** | **Description according to the ICD** | **Chapter of the ICD** | **Depression** | | | | | | | |
| --- | --- | --- | --- | --- | --- | --- | --- | --- | --- | --- |
|  |  |  | **mild** | |  | **moderate** | |  | **severe** | |
|  |  |  | **P (%)** | **PR** |  | **P (%)** | **PR** |  | **P (%)** | **PR** |
| A00-A09 | Intestinal infectious diseases | I - Certain infectious and parasitic diseases | 9,02 | 1,54 |  | 9,82 | 1,62 |  | 9,30 | 1,58 |
| A15-A19 | Tuberculosis |  | 0,16 | 1,68 |  | 0,15 | 1,67 |  | 0,18 | 1,96 |
| A20-A28 | Certain zoonotic bacterial diseases |  | 0,20 | 1,28 |  | 0,20 | 1,33 |  | 0,20 | 1,31 |
| A30-A49 | Other bacterial diseases |  | 2,15 | 1,50 |  | 2,12 | 1,52 |  | 2,37 | 1,68 |
| A50-A64 | Infections with a predominantly sexual mode of transmission |  | 0,87 | 1,89 |  | 0,91 | 1,90 |  | 0,91 | 1,98 |
| A65-A69 | Other spirochaetal diseases |  | 0,84 | 1,58 |  | 0,85 | 1,66 |  | 0,87 | 1,68 |
| A70-A74 | Other diseases caused by chlamydiae |  | 0,13 | 1,78 |  | 0,14 | 1,89 |  | 0,12 | 1,88 |
| A75-A79 | Rickettsioses |  | 0,01 | 1,75 |  | 0,01 | 1,75 |  | 0,01 | 2,00 |
| A80-A89 | Viral infections of the central nervous system |  | 0,10 | 2,06 |  | 0,10 | 2,10 |  | 0,11 | 2,18 |
| A92-A99 | Arthropod-borne viral fevers and viral haemorrhagic fevers |  | 0,01 | 1,40 |  | 0,01 | 1,20 |  | 0,01 | 1,17 |
| B00-B09 | Viral infections characterized by skin and mucous membrane lesions |  | 4,90 | 1,45 |  | 4,78 | 1,43 |  | 4,75 | 1,43 |
| B15-B19 | Viral hepatitis |  | 0,95 | 2,05 |  | 1,07 | 2,27 |  | 1,30 | 2,63 |
| B20-B24 | Human immunodeficiency virus [HIV] disease |  | 0,22 | 2,68 |  | 0,23 | 2,59 |  | 0,28 | 2,94 |
| B25-B34 | Other viral diseases |  | 3,92 | 1,38 |  | 4,08 | 1,35 |  | 3,53 | 1,20 |
| B35-B49 | Mycoses |  | 9,06 | 1,57 |  | 8,83 | 1,55 |  | 9,32 | 1,63 |
| B50-B64 | Protozoal diseases |  | 0,06 | 1,57 |  | 0,05 | 1,51 |  | 0,06 | 1,60 |
| B65-B83 | Helminthiases |  | 0,29 | 1,42 |  | 0,28 | 1,30 |  | 0,26 | 1,26 |
| B85-B89 | Pediculosis, acariasis and other infestations |  | 0,52 | 1,57 |  | 0,58 | 1,63 |  | 0,61 | 1,81 |
| B90-B94 | Sequelae of infectious and parasitic diseases |  | 0,20 | 1,61 |  | 0,19 | 1,72 |  | 0,22 | 1,90 |
| B95-B98 | Bacterial, viral and other infectious agents |  | 1,25 | 1,75 |  | 1,24 | 1,79 |  | 1,33 | 1,91 |
| B99-B99 | Other infectious diseases |  | 2,14 | 1,46 |  | 2,09 | 1,40 |  | 2,11 | 1,44 |
| C00-C14 | Malignant neoplasms of lip, oral cavity and pharynx | II - Neoplasms | 0,24 | 1,48 |  | 0,23 | 1,57 |  | 0,24 | 1,55 |
| C15-C26 | Malignant neoplasms of digestive organs |  | 1,55 | 1,35 |  | 1,38 | 1,40 |  | 1,42 | 1,37 |
| C30-C39 | Malignant neoplasms of respiratory and intrathoracic organs |  | 0,54 | 1,48 |  | 0,53 | 1,65 |  | 0,58 | 1,65 |
| C40-C41 | Malignant neoplasms of bone and articular cartilage |  | 0,06 | 1,93 |  | 0,06 | 2,00 |  | 0,07 | 2,10 |
| C43-C44 | Melanoma and other malignant neoplasms of skin |  | 3,55 | 1,23 |  | 2,95 | 1,18 |  | 2,93 | 1,12 |
| C45-C49 | Malignant neoplasms of mesothelial and soft tissue |  | 0,18 | 1,72 |  | 0,16 | 1,69 |  | 0,18 | 1,76 |
| C50-C50 | Malignant neoplasm of breast |  | 3,38 | 1,91 |  | 2,83 | 1,69 |  | 2,82 | 1,67 |
| C51-C58 | Malignant neoplasms of female genital organs |  | 0,95 | 1,75 |  | 0,84 | 1,59 |  | 0,86 | 1,63 |
| C60-C63 | Malignant neoplasms of male genital organs |  | 1,63 | 1,60 |  | 1,13 | 1,46 |  | 1,21 | 1,44 |
| C64-C68 | Malignant neoplasms of urinary tract |  | 1,05 | 1,55 |  | 0,83 | 1,48 |  | 0,89 | 1,51 |
| C69-C72 | Malignant neoplasms of eye, brain and other parts of central nervous system |  | 0,20 | 1,98 |  | 0,21 | 2,12 |  | 0,22 | 2,14 |
| C73-C75 | Malignant neoplasms of thyroid and other endocrine glands |  | 0,31 | 1,63 |  | 0,31 | 1,70 |  | 0,32 | 1,71 |
| C76-C80 | Malignant neoplasms of ill-defined, secondary and unspecified sites |  | 1,94 | 1,72 |  | 1,64 | 1,66 |  | 1,71 | 1,64 |
| C81-C96 | Malignant neoplasms, stated or presumed to be primary, of lymphoid, haematopoietic and related tissue |  | 1,03 | 1,50 |  | 0,91 | 1,48 |  | 0,94 | 1,48 |
| C97-C97 | Malignant neoplasms of independent (primary) multiple sites |  | 0,02 | 1,58 |  | 0,02 | 1,64 |  | 0,02 | 2,00 |
| D00-D09 | In situ neoplasms |  | 2,26 | 1,33 |  | 1,79 | 1,22 |  | 1,74 | 1,15 |
| D10-D36 | Benign neoplasms |  | 20,81 | 1,30 |  | 19,85 | 1,24 |  | 19,30 | 1,19 |
| D37-D48 | Neoplasms of uncertain or unknown behaviour |  | 5,12 | 1,51 |  | 4,73 | 1,47 |  | 4,94 | 1,49 |
| D50-D53 | Nutritional anaemias | III - Diseases of the blood and blood-forming organs and certain disorders involving the immune mechanism | 5,61 | 1,64 |  | 5,65 | 1,70 |  | 6,16 | 1,89 |
| D55-D59 | Haemolytic anaemias |  | 0,29 | 1,65 |  | 0,28 | 1,61 |  | 0,33 | 1,89 |
| D60-D64 | Aplastic and other anaemias |  | 3,87 | 1,62 |  | 3,43 | 1,57 |  | 3,93 | 1,78 |
| D65-D69 | Coagulation defects, purpura and other haemorrhagic conditions |  | 3,04 | 1,55 |  | 2,77 | 1,57 |  | 2,89 | 1,62 |
| D70-D77 | Other diseases of blood and blood-forming organs |  | 1,84 | 1,63 |  | 1,85 | 1,67 |  | 2,05 | 1,79 |
| D80-D90 | Certain disorders involving the immune mechanism |  | 1,43 | 1,93 |  | 1,39 | 1,86 |  | 1,45 | 1,91 |
| E00-E07 | Disorders of thyroid gland | IV - Endocrine, nutritional and metabolic diseases | 30,2 | 1,39 |  | 29,9 | 1,40 |  | 30,8 | 1,43 |
| E10-E14 | Diabetes mellitus |  | 18,3 | 1,33 |  | 17,0 | 1,39 |  | 19,0 | 1,50 |
| E15-E16 | Other disorders of glucose regulation and pancreatic internal secretion |  | 0,35 | 2,12 |  | 0,35 | 2,28 |  | 0,40 | 2,52 |
| E20-E35 | Disorders of other endocrine glands |  | 5,08 | 1,75 |  | 4,82 | 1,66 |  | 5,02 | 1,74 |
| E40-E46 | Malnutrition |  | 0,27 | 2,02 |  | 0,28 | 2,36 |  | 0,37 | 3,09 |
| E50-E64 | Other nutritional deficiencies |  | 8,53 | 1,85 |  | 8,99 | 2,04 |  | 9,64 | 2,18 |
| E65-E68 | Obesity and other hyperalimentation |  | 19,0 | 1,52 |  | 19,4 | 1,58 |  | 21,2 | 1,69 |
| E70-E90 | Metabolic disorders |  | 40,4 | 1,31 |  | 38,4 | 1,33 |  | 40,6 | 1,35 |
| F00-F09 | Organic, including symptomatic, mental disorders | V - Mental and behavioural disorders | 7,85 | 2,23 |  | 8,13 | 2,76 |  | 10,81 | 3,60 |
| F10-F19 | Mental and behavioural disorders due to psychoactive substance use |  | 12,5 | 1,95 |  | 15,9 | 2,38 |  | 20,11 | 2,89 |
| F20-F29 | Schizophrenia, schizotypal and delusional disorders |  | 2,39 | 3,54 |  | 3,06 | 4,57 |  | 7,07 | 10,22 |
| F40-F48 | Neurotic, stress-related and somatoform disorders |  | 52,4 | 3,29 |  | 61,2 | 3,84 |  | 65,5 | 4,18 |
| F50-F59 | Behavioural syndromes associated with physiological disturbances and physical factors |  | 9,10 | 3,42 |  | 10,8 | 4,29 |  | 12,9 | 4,95 |
| F60-F69 | Disorders of adult personality and behaviour |  | 6,49 | 7,47 |  | 9,43 | 11,0 |  | 14,3 | 17,1 |
| F70-F79 | Mental retardation |  | 0,87 | 1,63 |  | 0,80 | 1,41 |  | 1,10 | 1,98 |
| F80-F89 | Disorders of psychological development |  | 0,69 | 2,22 |  | 0,72 | 2,34 |  | 0,80 | 2,97 |
| F90-F98 | Behavioural and emotional disorders with onset usually occurring in childhood and adolescence |  | 2,35 | 4,00 |  | 2,71 | 4,84 |  | 3,20 | 6,10 |
| F99-F99 | Unspecified mental disorder |  | 1,06 | 3,52 |  | 1,19 | 3,96 |  | 1,54 | 5,26 |
| G00-G09 | Inflammatory diseases of the central nervous system | VI - Diseases of the nervous system | 0,32 | 2,52 |  | 0,34 | 2,72 |  | 0,37 | 2,92 |
| G10-G14 | Systemic atrophies primarily affecting the central nervous system |  | 0,26 | 2,36 |  | 0,27 | 2,60 |  | 0,32 | 3,00 |
| G20-G26 | Extrapyramidal and movement disorders |  | 5,67 | 2,78 |  | 5,99 | 3,33 |  | 7,47 | 4,02 |
| G30-G32 | Other degenerative diseases of the nervous system |  | 2,03 | 2,16 |  | 2,30 | 2,95 |  | 3,04 | 3,81 |
| G35-G37 | Demyelinating diseases of the central nervous system |  | 1,10 | 3,02 |  | 1,11 | 2,93 |  | 1,02 | 2,72 |
| G40-G47 | Episodic and paroxysmal disorders |  | 28,2 | 2,41 |  | 31,1 | 2,68 |  | 34,2 | 2,94 |
| G50-G59 | Nerve, nerve root and plexus disorders |  | 12,4 | 2,03 |  | 13,1 | 2,19 |  | 14,7 | 2,41 |
| G60-G64 | Polyneuropathies and other disorders of the peripheral nervous system |  | 9,17 | 2,14 |  | 8,69 | 2,34 |  | 9,91 | 2,57 |
| G70-G73 | Diseases of myoneural junction and muscle |  | 0,57 | 2,35 |  | 0,53 | 2,32 |  | 0,64 | 2,72 |
| G80-G83 | Cerebral palsy and other paralytic syndromes |  | 2,86 | 2,11 |  | 3,00 | 2,42 |  | 3,16 | 2,49 |
| G90-G99 | Other disorders of the nervous system |  | 2,92 | 2,49 |  | 3,08 | 2,79 |  | 3,48 | 3,08 |
| H00-H06 | Disorders of eyelid, lacrimal system and orbit | VII - Diseases of the eye and adnexa | 10,5 | 1,38 |  | 9,54 | 1,34 |  | 9,45 | 1,31 |
| H10-H13 | Disorders of conjunctiva |  | 7,52 | 1,35 |  | 7,17 | 1,32 |  | 7,03 | 1,29 |
| H15-H22 | Disorders of sclera, cornea, iris and ciliary body |  | 4,51 | 1,32 |  | 3,98 | 1,26 |  | 3,92 | 1,22 |
| H25-H28 | Disorders of lens |  | 15,5 | 1,22 |  | 12,8 | 1,19 |  | 13,1 | 1,18 |
| H30-H36 | Disorders of choroid and retina |  | 10,9 | 1,25 |  | 9,14 | 1,21 |  | 9,18 | 1,19 |
| H40-H42 | Glaucoma |  | 6,94 | 1,22 |  | 5,93 | 1,17 |  | 5,95 | 1,15 |
| H43-H45 | Disorders of vitreous body and globe |  | 4,52 | 1,29 |  | 3,79 | 1,23 |  | 3,73 | 1,18 |
| H46-H48 | Disorders of optic nerve and visual pathways |  | 3,17 | 1,27 |  | 2,74 | 1,21 |  | 2,75 | 1,17 |
| H49-H52 | Disorders of ocular muscles, binocular movement, accommodation and refraction |  | 28,6 | 1,23 |  | 26,0 | 1,20 |  | 25,8 | 1,16 |
| H53-H54 | Visual disturbances and blindness |  | 9,41 | 1,38 |  | 8,70 | 1,37 |  | 8,99 | 1,38 |
| H55-H59 | Other disorders of eye and adnexa |  | 2,21 | 1,36 |  | 1,99 | 1,33 |  | 2,01 | 1,31 |
| H60-H62 | Diseases of external ear | VIII - Diseases of the ear and mastoid process | 12,0 | 1,34 |  | 11,3 | 1,34 |  | 11,7 | 1,34 |
| H65-H75 | Diseases of middle ear and mastoid |  | 4,81 | 1,39 |  | 4,82 | 1,41 |  | 4,86 | 1,42 |
| H80-H83 | Diseases of inner ear |  | 4,35 | 2,04 |  | 4,22 | 2,12 |  | 4,54 | 2,22 |
| H90-H95 | Other disorders of ear |  | 16,0 | 1,56 |  | 15,6 | 1,66 |  | 16,5 | 1,71 |
| I00-I02 | Acute rheumatic fever | IX - Diseases of the circulatory system | 0,09 | 1,89 |  | 0,09 | 1,91 |  | 0,11 | 2,46 |
| I05-I09 | Chronic rheumatic heart diseases |  | 2,62 | 1,46 |  | 2,12 | 1,41 |  | 2,15 | 1,38 |
| I10-I15 | Hypertensive diseases |  | 47,7 | 1,21 |  | 45,3 | 1,23 |  | 47,7 | 1,25 |
| I20-I25 | Ischaemic heart diseases |  | 13,2 | 1,48 |  | 11,7 | 1,53 |  | 12,9 | 1,62 |
| I26-I28 | Pulmonary heart disease and diseases of pulmonary circulation |  | 1,61 | 1,46 |  | 1,43 | 1,53 |  | 1,54 | 1,61 |
| I30-I52 | Other forms of heart disease |  | 23,1 | 1,40 |  | 20,8 | 1,44 |  | 21,7 | 1,45 |
| I60-I69 | Cerebrovascular diseases |  | 11,1 | 1,66 |  | 10,4 | 1,82 |  | 11,2 | 1,87 |
| I70-I79 | Diseases of arteries, arterioles and capillaries |  | 11,8 | 1,52 |  | 10,0 | 1,49 |  | 10,6 | 1,50 |
| I80-I89 | Diseases of veins, lymphatic vessels and lymph nodes, not elsewhere classified |  | 20,2 | 1,36 |  | 19,0 | 1,36 |  | 19,0 | 1,34 |
| I95-I99 | Other and unspecified disorders of the circulatory system |  | 6,22 | 1,86 |  | 5,98 | 1,81 |  | 6,37 | 1,96 |
| J00-J06 | Acute upper respiratory infections | X - Diseases of the respiratory system | 27,5 | 1,30 |  | 28,6 | 1,28 |  | 26,0 | 1,20 |
| J09-J18 | Influenza and pneumonia |  | 2,75 | 1,50 |  | 2,79 | 1,55 |  | 3,17 | 1,73 |
| J20-J22 | Other acute lower respiratory infections |  | 7,95 | 1,45 |  | 8,63 | 1,54 |  | 8,90 | 1,57 |
| J30-J39 | Other diseases of upper respiratory tract |  | 20,5 | 1,49 |  | 20,8 | 1,48 |  | 20,1 | 1,43 |
| J40-J47 | Chronic lower respiratory diseases |  | 23,8 | 1,53 |  | 24,5 | 1,60 |  | 26,1 | 1,67 |
| J60-J70 | Lung diseases due to external agents |  | 0,28 | 1,62 |  | 0,27 | 1,78 |  | 0,32 | 1,95 |
| J80-J84 | Other respiratory diseases principally affecting the interstitium |  | 0,47 | 1,64 |  | 0,44 | 1,76 |  | 0,50 | 1,89 |
| J85-J86 | Suppurative and necrotic conditions of lower respiratory tract |  | 0,04 | 1,75 |  | 0,04 | 1,78 |  | 0,05 | 2,26 |
| J90-J94 | Other diseases of pleura |  | 0,49 | 1,48 |  | 0,46 | 1,62 |  | 0,51 | 1,70 |
| J95-J99 | Other diseases of the respiratory system |  | 4,04 | 1,64 |  | 3,93 | 1,62 |  | 4,12 | 1,67 |
| K00-K14 | Diseases of oral cavity, salivary glands and jaws | XI - Diseases of the digestive system | 3,77 | 1,79 |  | 3,90 | 1,85 |  | 4,19 | 1,99 |
| K20-K31 | Diseases of oesophagus, stomach and duodenum |  | 26,4 | 1,85 |  | 27,1 | 1,97 |  | 29,5 | 2,08 |
| K35-K38 | Diseases of appendix |  | 0,34 | 1,58 |  | 0,34 | 1,55 |  | 0,37 | 1,70 |
| K40-K46 | Hernia |  | 6,63 | 1,63 |  | 6,32 | 1,67 |  | 6,93 | 1,73 |
| K50-K52 | Noninfective enteritis and colitis |  | 5,55 | 1,68 |  | 5,95 | 1,74 |  | 6,02 | 1,79 |
| K55-K64 | Other diseases of intestines |  | 20,0 | 1,66 |  | 19,4 | 1,71 |  | 20,6 | 1,76 |
| K65-K67 | Diseases of peritoneum |  | 0,76 | 2,07 |  | 0,81 | 2,21 |  | 0,86 | 2,33 |
| K70-K77 | Diseases of liver |  | 11,3 | 1,55 |  | 11,1 | 1,59 |  | 12,5 | 1,70 |
| K80-K87 | Disorders of gallbladder, biliary tract and pancreas |  | 7,37 | 1,45 |  | 7,08 | 1,49 |  | 7,72 | 1,57 |
| K90-K93 | Other diseases of the digestive system |  | 2,41 | 1,78 |  | 2,48 | 1,89 |  | 2,58 | 1,95 |
| L00-L08 | Infections of the skin and subcutaneous tissue | XII - Diseases of the skin and subcutaneous tissue | 4,29 | 1,54 |  | 4,42 | 1,59 |  | 4,78 | 1,72 |
| L10-L14 | Bullous disorders |  | 0,16 | 1,61 |  | 0,15 | 1,66 |  | 0,18 | 2,06 |
| L20-L30 | Dermatitis and eczema |  | 18,7 | 1,50 |  | 18,3 | 1,50 |  | 18,8 | 1,54 |
| L40-L45 | Papulosquamous disorders |  | 4,50 | 1,43 |  | 4,50 | 1,44 |  | 4,66 | 1,46 |
| L50-L54 | Urticaria and erythema |  | 1,67 | 1,61 |  | 1,73 | 1,67 |  | 1,76 | 1,71 |
| L55-L59 | Radiation-related disorders of the skin and subcutaneous tissue |  | 3,36 | 1,27 |  | 2,74 | 1,22 |  | 2,62 | 1,12 |
| L60-L75 | Disorders of skin appendages |  | 11,4 | 1,47 |  | 11,2 | 1,45 |  | 10,9 | 1,44 |
| L80-L99 | Other disorders of the skin and subcutaneous tissue |  | 11,1 | 1,40 |  | 10,4 | 1,39 |  | 10,7 | 1,41 |
| M00-M03 | Infectious arthropathies | XIII - Diseases of the musculoskeletal system and connective tissue | 0,28 | 1,73 |  | 0,28 | 1,75 |  | 0,29 | 1,76 |
| M05-M14 | Inflammatory polyarthropathies |  | 9,00 | 1,51 |  | 8,62 | 1,55 |  | 9,29 | 1,62 |
| M15-M19 | Arthrosis |  | 30,1 | 1,45 |  | 28,5 | 1,50 |  | 29,8 | 1,51 |
| M20-M25 | Other joint disorders |  | 25,2 | 1,48 |  | 25,1 | 1,48 |  | 25,4 | 1,48 |
| M30-M36 | Systemic connective tissue disorders |  | 4,11 | 1,66 |  | 3,77 | 1,68 |  | 3,76 | 1,66 |
| M40-M43 | Deforming dorsopathies |  | 18,8 | 1,67 |  | 18,8 | 1,71 |  | 19,4 | 1,73 |
| M45-M49 | Spondylopathies |  | 23,5 | 1,78 |  | 22,9 | 1,84 |  | 24,5 | 1,89 |
| M50-M54 | Other dorsopathies |  | 53,3 | 1,54 |  | 53,9 | 1,56 |  | 55,1 | 1,57 |
| M60-M63 | Disorders of muscles |  | 8,68 | 1,78 |  | 8,82 | 1,81 |  | 9,01 | 1,83 |
| M65-M68 | Disorders of synovium and tendon |  | 4,70 | 1,48 |  | 4,84 | 1,50 |  | 4,88 | 1,47 |
| M70-M79 | Other soft tissue disorders |  | 29,3 | 1,57 |  | 30,2 | 1,61 |  | 31,2 | 1,61 |
| M80-M85 | Disorders of bone density and structure |  | 10,2 | 1,62 |  | 9,13 | 1,66 |  | 9,48 | 1,69 |
| M86-M90 | Other osteopathies |  | 1,41 | 1,76 |  | 1,44 | 1,86 |  | 1,56 | 1,96 |
| M91-M94 | Chondropathies |  | 3,41 | 1,73 |  | 3,49 | 1,79 |  | 3,81 | 1,85 |
| M95-M99 | Other disorders of the musculoskeletal system and connective tissue |  | 15,7 | 1,66 |  | 16,3 | 1,68 |  | 16,1 | 1,64 |
| N00-N08 | Glomerular diseases | XIV - Diseases of the genitourinary system | 2,71 | 1,57 |  | 2,40 | 1,59 |  | 2,74 | 1,74 |
| N10-N16 | Renal tubulo-interstitial diseases |  | 1,68 | 1,65 |  | 1,56 | 1,61 |  | 1,73 | 1,82 |
| N17-N19 | Renal failure |  | 8,82 | 1,48 |  | 7,78 | 1,54 |  | 8,47 | 1,62 |
| N20-N23 | Urolithiasis |  | 2,85 | 1,56 |  | 2,64 | 1,52 |  | 2,87 | 1,58 |
| N25-N29 | Other disorders of kidney and ureter |  | 4,50 | 1,47 |  | 3,86 | 1,42 |  | 4,16 | 1,46 |
| N30-N39 | Other diseases of urinary system |  | 17,8 | 1,70 |  | 16,8 | 1,70 |  | 17,8 | 1,80 |
| N40-N51 | Diseases of male genital organs |  | 8,44 | 1,44 |  | 7,01 | 1,41 |  | 7,91 | 1,45 |
| N60-N64 | Disorders of breast |  | 6,86 | 1,43 |  | 6,56 | 1,31 |  | 6,07 | 1,26 |
| N70-N77 | Inflammatory diseases of female pelvic organs |  | 7,71 | 1,52 |  | 7,33 | 1,39 |  | 6,86 | 1,40 |
| N80-N98 | Noninflammatory disorders of female genital tract |  | 35,3 | 1,26 |  | 34,0 | 1,16 |  | 30,9 | 1,12 |
| N99-N99 | Other disorders of the genitourinary system |  | 0,54 | 1,78 |  | 0,51 | 1,75 |  | 0,53 | 1,76 |
| O00-O08 | Pregnancy with abortive outcome | XV - Pregnancy, childbirth and the puerperium | 0,63 | 1,86 |  | 0,48 | 1,39 |  | 0,41 | 1,43 |
| O09-O09 | Duration of pregnancy |  | 1,54 | 1,08 |  | 1,14 | 0,78 |  | 0,90 | 0,76 |
| O10-O16 | Oedema, proteinuria and hypertensive disorders in pregnancy, childbirth and the puerperium |  | 0,24 | 1,24 |  | 0,17 | 0,84 |  | 0,14 | 0,83 |
| O20-O29 | Other maternal disorders predominantly related to pregnancy |  | 1,56 | 1,19 |  | 1,15 | 0,86 |  | 0,91 | 0,83 |
| O30-O48 | Maternal care related to the fetus and amniotic cavity and possible delivery problems |  | 0,97 | 1,04 |  | 0,68 | 0,71 |  | 0,53 | 0,67 |
| O60-O75 | Complications of labour and delivery |  | 0,39 | 1,09 |  | 0,26 | 0,70 |  | 0,21 | 0,69 |
| O80-O82 | Delivery |  | 0,26 | 0,99 |  | 0,21 | 0,75 |  | 0,18 | 0,76 |
| O85-O92 | Complications predominantly related to the puerperium |  | 0,36 | 1,02 |  | 0,28 | 0,76 |  | 0,22 | 0,75 |
| O94-O99 | Other obstetric conditions, not elsewhere classified |  | 0,60 | 1,19 |  | 0,45 | 0,87 |  | 0,37 | 0,88 |
| Q00-Q07 | Congenital malformations of the nervous system | XVII - Congenital malformations, deformations and chromosomal abnormalities | 0,15 | 1,97 |  | 0,15 | 1,85 |  | 0,16 | 2,04 |
| Q10-Q18 | Congenital malformations of eye, ear, face and neck |  | 0,61 | 1,27 |  | 0,54 | 1,21 |  | 0,54 | 1,19 |
| Q20-Q28 | Congenital malformations of the circulatory system |  | 0,82 | 1,49 |  | 0,81 | 1,56 |  | 0,81 | 1,57 |
| Q30-Q34 | Congenital malformations of the respiratory system |  | 0,05 | 1,81 |  | 0,05 | 1,84 |  | 0,05 | 1,85 |
| Q35-Q37 | Cleft lip and cleft palate |  | 0,03 | 1,32 |  | 0,03 | 1,50 |  | 0,03 | 1,48 |
| Q38-Q45 | Other congenital malformations of the digestive system |  | 0,68 | 1,62 |  | 0,63 | 1,62 |  | 0,70 | 1,70 |
| Q50-Q56 | Congenital malformations of genital organs |  | 0,31 | 1,59 |  | 0,28 | 1,40 |  | 0,29 | 1,50 |
| Q60-Q64 | Congenital malformations of the urinary system |  | 2,84 | 1,46 |  | 2,41 | 1,40 |  | 2,57 | 1,43 |
| Q65-Q79 | Congenital malformations and deformations of the musculoskeletal system |  | 13,1 | 1,46 |  | 12,9 | 1,46 |  | 12,9 | 1,42 |
| Q80-Q89 | Other congenital malformations |  | 1,17 | 1,50 |  | 1,15 | 1,49 |  | 1,15 | 1,51 |
| Q90-Q99 | Chromosomal abnormalities, not elsewhere classified |  | 0,12 | 1,11 |  | 0,10 | 0,91 |  | 0,11 | 0,96 |
| S00-S09 | Injuries to the head | XIX - Injury, poisoning and certain other consequences of external causes | 2,96 | 1,51 |  | 3,08 | 1,65 |  | 3,48 | 1,86 |
| S10-S19 | Injuries to the neck |  | 0,86 | 1,77 |  | 0,96 | 1,94 |  | 1,06 | 2,17 |
| S20-S29 | Injuries to the thorax |  | 2,18 | 1,56 |  | 2,27 | 1,70 |  | 2,49 | 1,81 |
| S30-S39 | Injuries to the abdomen, lower back, lumbar spine and pelvis |  | 2,11 | 1,65 |  | 2,15 | 1,83 |  | 2,38 | 1,98 |
| S40-S49 | Injuries to the shoulder and upper arm |  | 2,10 | 1,44 |  | 2,11 | 1,53 |  | 2,31 | 1,62 |
| S50-S59 | Injuries to the elbow and forearm |  | 1,58 | 1,37 |  | 1,67 | 1,53 |  | 1,86 | 1,70 |
| S60-S69 | Injuries to the wrist and hand |  | 2,91 | 1,36 |  | 3,13 | 1,45 |  | 3,27 | 1,51 |
| S70-S79 | Injuries to the hip and thigh |  | 1,54 | 1,45 |  | 1,53 | 1,58 |  | 1,74 | 1,76 |
| S80-S89 | Injuries to the knee and lower leg |  | 3,94 | 1,36 |  | 4,09 | 1,42 |  | 4,33 | 1,48 |
| S90-S99 | Injuries to the ankle and foot |  | 3,34 | 1,36 |  | 3,61 | 1,44 |  | 3,64 | 1,47 |
| T00-T07 | Injuries involving multiple body regions |  | 0,98 | 1,60 |  | 0,99 | 1,75 |  | 1,19 | 2,06 |
| T08-T14 | Injuries to unspecified part of trunk, limb or body region |  | 9,12 | 1,41 |  | 9,06 | 1,46 |  | 9,64 | 1,53 |
| T15-T19 | Effects of foreign body entering through natural orifice |  | 1,12 | 1,30 |  | 1,02 | 1,23 |  | 1,08 | 1,26 |
| T20-T25 | Burns and corrosions of external body surface, specified by site |  | 0,24 | 1,54 |  | 0,27 | 1,69 |  | 0,30 | 1,92 |
| T26-T28 | Burns and corrosions confined to eye and internal organs |  | 0,03 | 1,46 |  | 0,03 | 1,50 |  | 0,03 | 1,52 |
| T29-T32 | Burns and corrosions of multiple and unspecified body regions |  | 0,36 | 1,57 |  | 0,38 | 1,62 |  | 0,43 | 1,83 |
| T33-T35 | Frostbite |  | 0,01 | 1,80 |  | 0,01 | 1,80 |  | 0,01 | 2,00 |
| T36-T50 | Poisoning by drugs, medicaments and biological substances |  | 0,15 | 2,01 |  | 0,18 | 2,58 |  | 0,30 | 4,29 |
| T51-T65 | Toxic effects of substances chiefly nonmedicinal as to source |  | 1,02 | 1,43 |  | 1,10 | 1,54 |  | 1,18 | 1,63 |
| T66-T78 | Other and unspecified effects of external causes |  | 8,51 | 1,67 |  | 8,45 | 1,64 |  | 8,53 | 1,66 |
| T79-T79 | Certain early complications of trauma |  | 1,49 | 1,50 |  | 1,49 | 1,56 |  | 1,66 | 1,70 |
| T80-T88 | Complications of surgical and medical care, not elsewhere classified |  | 8,51 | 1,64 |  | 8,34 | 1,72 |  | 8,79 | 1,78 |
| T89-T89 | Other complications of trauma, not elsewhere classified |  | 0,58 | 1,63 |  | 0,54 | 1,63 |  | 0,61 | 1,81 |
| T90-T98 | Sequelae of injuries, of poisoning and of other consequences of external causes |  | 0,61 | 1,72 |  | 0,64 | 1,92 |  | 0,73 | 2,14 |
| V01-X59 | Accidents | XX - External causes of morbidity and mortality | 0,23 | 1,39 |  | 0,26 | 1,51 |  | 0,26 | 1,60 |
| X60-X84 | Intentional self-harm |  | 0,03 | 13,50 |  | 0,08 | 37,50 |  | 0,19 | 93,50 |
| X85-Y09 | Assault |  | 0,02 | 1,88 |  | 0,02 | 2,38 |  | 0,03 | 3,71 |
| Y10-Y34 | Event of undetermined intent |  | 0,00 | 2,00 |  | 0,00 | 2,00 |  | 0,01 | 3,00 |
| Y40-Y84 | Complications of medical and surgical care |  | 0,22 | 1,95 |  | 0,21 | 1,95 |  | 0,22 | 2,02 |

ICD, International Statistical Classification of Diseases and Related Health Problems.
The prevalence ratio is defined as the ratio of the prevalence of the respective diagnosis group among depression cases to the prevalence among age-, sex- and region-matched controls.
