# Supplementary material for: Mental and somatic comorbidity of depression: a comprehensive cross-sectional analysis of 202 diagnosis groups using German nationwide ambulatory claims data
Source: BMC Psychiatry. 2020 Mar 30;20:142. doi: 10.1186/s12888-020-02546-8 (PMC7106695; doi:10.1186/s12888-020-02546-8)
Supplement: Supplementary file 2 — Additional file 2:Table A2. Age-adjusted prevalence (%) and prevalence ratios (PR) for the 20 most prevalent comorbidities by sex among individuals with mild depressive disorder. This supplemental table provides age-adjusted prevalence and prevalence ratios for the 20 most prevalent comorbidities by sex among individuals with mild depressive disorder. [file 12888_2020_2546_MOESM2_ESM.docx]

Table A2. Age-adjusted prevalence (%) and prevalence ratios (PR) for the 20 most prevalent comorbidities by sex among individuals with mild depressive disorder

| **mild depressive disorder** | | | | | | |
| --- | --- | --- | --- | --- | --- | --- |
|  | **Men** | | | **Women** | | |
| **ICD group** | **Rank among cases** | **prevalence among cases (%)** | **PR** | **Rank among cases** | **prevalence among cases (%)** | **PR** |
| D10-D36 | 19 | 17.6 | 1.28 | 16 | 22.6 | 1.30 |
| E00-E07 | 25 | 15.9 | 1.51 | 5 | 37.2 | 1.37 |
| E65-E68 | 21 | 17.3 | 1.49 | 22 | 19.9 | 1.53 |
| E70-E90 | 4 | 43.4 | 1.29 | 4 | 38.8 | 1.33 |
| F10-F19 | 22 | 16.9 | 1.94 | 38 | 10.2 | 1.95 |
| F40-F48 | 3 | 46.1 | 4.24 | 1 | 55.4 | 3.02 |
| G40-G47 | 6 | 26.7 | 2.54 | 9 | 28.9 | 2.35 |
| H49-H52 | 9 | 25.6 | 1.22 | 8 | 30.1 | 1.24 |
| I10-I15 | 1 | 51.3 | 1.22 | 3 | 45.8 | 1.20 |
| I30-I52 | 10 | 25.6 | 1.37 | 17 | 21.9 | 1.42 |
| I80-I89 | 30 | 13.6 | 1.36 | 15 | 23.3 | 1.37 |
| J00-J06 | 11 | 25.4 | 1.21 | 10 | 28.5 | 1.34 |
| J30-J39 | 17 | 19.5 | 1.44 | 18 | 21.1 | 1.52 |
| J40-J47 | 12 | 24.4 | 1.47 | 14 | 23.6 | 1.56 |
| K20-K31 | 7 | 26.6 | 1.81 | 12 | 26.3 | 1.87 |
| K55-K64 | 16 | 20.0 | 1.59 | 21 | 20.0 | 1.70 |
| M15-M19 | 5 | 26.8 | 1.46 | 6 | 31.6 | 1.45 |
| M20-M25 | 15 | 21.6 | 1.41 | 11 | 26.9 | 1.50 |
| M45-M49 | 13 | 22.3 | 1.77 | 13 | 24.1 | 1.78 |
| M50-M54 | 2 | 50.0 | 1.50 | 2 | 55.0 | 1.56 |
| M70-M79 | 8 | 26.1 | 1.46 | 7 | 30.9 | 1.62 |
|  | | | | | | |
| **severe depressive disorder** | | | | | | |
|  | **Men** | | | **Women** | | |
| **ICD group** | **Rank among cases** | **prevalence among cases (%)** | **PR** | **Rank among cases** | **prevalence among cases (%)** | **PR** |
| D10-D36 | 25 | 16.1 | 1.19 | 17 | 21.3 | 1.19 |
| E00-E07 | 22 | 16.5 | 1.61 | 5 | 38.4 | 1.39 |
| E65-E68 | 20 | 18.2 | 1.57 | 15 | 22.8 | 1.75 |
| E70-E90 | 4 | 42.9 | 1.30 | 4 | 39.3 | 1.38 |
| F10-F19 | 10 | 25.2 | 2.73 | 26 | 17.2 | 3.04 |
| F40-F48 | 1 | 59.1 | 5.52 | 1 | 69.0 | 3.76 |
| G40-G47 | 5 | 32.5 | 3.12 | 6 | 35.3 | 2.86 |
| H49-H52 | 13 | 22.9 | 1.16 | 12 | 27.4 | 1.16 |
| I10-I15 | 3 | 50.8 | 1.24 | 3 | 45.9 | 1.26 |
| I30-I52 | 11 | 23.5 | 1.40 | 22 | 20.8 | 1.48 |
| I80-I89 | 33 | 12.8 | 1.34 | 16 | 22.1 | 1.34 |
| J00-J06 | 12 | 23.4 | 1.08 | 11 | 27.4 | 1.26 |
| J30-J39 | 18 | 18.7 | 1.36 | 21 | 20.9 | 1.47 |
| J40-J47 | 9 | 25.5 | 1.54 | 13 | 26.6 | 1.74 |
| K20-K31 | 6 | 28.7 | 1.96 | 9 | 30.0 | 2.15 |
| K55-K64 | 17 | 20.1 | 1.64 | 20 | 20.9 | 1.83 |
| M15-M19 | 8 | 25.8 | 1.48 | 8 | 31.7 | 1.52 |
| M20-M25 | 16 | 21.2 | 1.37 | 10 | 27.6 | 1.52 |
| M45-M49 | 14 | 22.5 | 1.82 | 14 | 25.5 | 1.92 |
| M50-M54 | 2 | 50.8 | 1.50 | 2 | 57.4 | 1.61 |
| M70-M79 | 7 | 26.6 | 1.44 | 7 | 33.7 | 1.70 |

Comorbidities were selected from the list of the 20 most prevalent comorbidities in the total population (see table 2). Sex-specific prevalence was age-adjusted using the joint age distribution of depression cases as reference, stratified by severity. The prevalence ratio is defined as the ratio of the prevalence of the respective diagnosis group among depression cases to the prevalence among controls.
ICD diagnosis groups (all 202 ICD diagnosis groups included in the present study are listed in table A2): D10-D36, Benign neoplasms; E00-E07, Disorders of thyroid gland; E65-E68, Obesity and other hyperalimentation; E70-E90, Metabolic disorders; F10-F19, Mental and behavioural disorders due to psychoactive substance use; F40-F48, Neurotic, stress-related and somatoform disorders; G40-G47, Episodic and paroxysmal disorders; H49-H52, Disorders of ocular muscles; I10-I15, Hypertension; I30-I52, Other forms of heart disease; I80-I89, Diseases of veins, lymphatic vessels and lymph nodes, not elsewhere classified; J00-J06, Acute upper respiratory infections; J30-J39, Other diseases of upper respiratory tract; J40-J47, Chronic lower respiratory diseases; K20-K31, Diseases of oesophagus, stomach and duodenum; K55-K64, Other diseases of intestines; M15-M19, Arthrosis; M20-M25, Other joint disorders; M45-M49, Spondylopathies; M50-M54, Other dorsopathies; M70-M79, Other soft tissue disorders.
